# Muscle satellite cells are a functionally heterogeneous population in both somite-derived and branchiomeric muscles

**DOI:** 10.1016/j.ydbio.2009.10.005

**Published:** 2010-01-01

**Authors:** Yusuke Ono, Luisa Boldrin, Paul Knopp, Jennifer E. Morgan, Peter S. Zammit

**Affiliations:** a King's College London, Randall Division of Cell and Molecular Biophysics, Guy's Campus, London SE1 1UL, UK; b Dubowitz Neuromuscular Centre, UCL Institute of Child Health, 30 Guilford Street, London, WC1N 1EH, UK

**Keywords:** Stem cell, Satellite cell, Skeletal muscle, Self-renewal, Differentiation, Pax7, MyoD, Heterogeneity, Aging, Somite, Branchial arch

## Abstract

Skeletal muscles of body and limb are derived from somites, but most head muscles originate from cranial mesoderm. The resident stem cells of muscle are satellite cells, which have the same embryonic origin as the muscle in which they reside. Here, we analysed satellite cells with a different ontology, comparing those of the extensor digitorum longus (EDL) of the limb with satellite cells from the masseter of the head. Satellite cell-derived myoblasts from MAS and EDL muscles had distinct gene expression profiles and masseter cells usually proliferated more and differentiated later than those from EDL. When transplanted, however, masseter-derived satellite cells regenerated limb muscles as efficiently as those from EDL. Clonal analysis showed that functional properties differed markedly between satellite cells: ranging from clones that proliferated extensively and gave rise to both differentiated and self-renewed progeny, to others that divided minimally before differentiating completely. Generally, masseter-derived clones were larger and took longer to differentiate than those from EDL. This distribution in cell properties was preserved in both EDL-derived and masseter-derived satellite cells from old mice, although clones were generally less proliferative. Satellite cells, therefore, are a functionally heterogeneous population, with many occupants of the niche exhibiting stem cell characteristics in both somite-derived and branchiomeric muscles.

## Introduction

Skeletal muscles of the trunk and limb are derived from myogenic precursor cells that migrate from certain somites, transitory mesoderm-derived structures that are formed in pairs on either side of the neural tube during embryonic development in vertebrates (Stockdale et al., 2000). In the head though, while tongue and neck muscle are formed from cells from the occipital somites, other head musculature arises from cranial mesoderm, located anterior to the somites (Noden and Francis-West, 2006). Cranial mesoderm is divided into two distinct domains, cranial paraxial mesoderm and lateral splanchnic mesoderm. Cells from these two sources migrate into the neighbouring branchial arches (also known as pharyngeal arches), to form the muscles that control jaw movement, facial expression, pharyngeal and laryngeal function (so called branchiomeric muscles) (Nathan et al., 2008). In contrast, extraocular muscles that control eye movement develop in situ in the periocular region, with input probably only from cranial paraxial mesoderm (Harel et al., 2009; Sambasivan et al., 2009).

Specification of pre-myogenic progenitors in the head involves distinct genetic networks to those that control formation of body/limb muscle (Noden and Francis-West, 2006; Shih et al., 2008). For example, mice null for the basic helix-loop-helix transcription factors *Myf5* and *MyoD* exhibit a complete absence of myogenesis, while double-knockout of the paired-box transcription factor *Pax3* and *Myf5* only have defects of body, but not head, musculature (Tajbakhsh et al., 1997). Unlike other muscles, the extraocular muscles lack an independent pathway to activate *MyoD* in the absence of both *Myf5* and *Mrf4* (Sambasivan et al., 2009). Other examples of the deployment of different regulatory networks include *MyoR/Tcf21* double-knockout mice that lack facial muscles including the masseter (MAS) (Lu et al., 2002); involvement of Pitx2 and Tbx1 in the specification of progenitor cells that give rise to branchiomeric muscles of the head muscles (Kelly et al., 2004; Shih et al., 2007); Wnt signalling acting as a positive regulator for myogenesis in trunk mesoderm, but blocking myogenesis in head (Tzahor et al., 2003); and Engrailed-2 being expressed in myoblasts in the first branchial arch and maintained in the muscles of mastication in the adult mouse (Degenhardt and Sassoon, 2001).

The resident stem cell of skeletal muscle is the ‘satellite cell,’ located between the basal lamina and the plasmalemma of a myofibre (Mauro, 1961). Satellite cells are responsible for providing myonuclei for postnatal muscle growth, then supplying new myonuclei for both routine homeostatic needs in adult skeletal muscle, and the sporadic demands for hypertrophy and repair (Kuang and Rudnicki, 2008; Zammit, 2008). Satellite cells of the body/limb arise from somites, in common with the muscle that they are associated with (Armand et al., 1983; Gros et al., 2005; Relaix et al., 2005; Schienda et al., 2006). It has recently been shown that this too is the case for the majority of satellite cells located in head muscles, which also originate from cranial mesoderm (Harel et al., 2009). These differences in the provenance and genetic control of head, compared to body/limb, musculature, may also endow the associated satellite cells with distinct properties, which could underlie observations that head muscles such as the MAS survive aging better (Norton et al., 2001) but regenerate poorly (Pavlath et al., 1998). In man, there is also evidence of poor recovery from damage of jaw musculature leading to temporo mandibular disorders (e.g., Pullinger and Monteiro, 1988), while certain muscular dystrophies affect head muscle severely, while they are relatively unaffected in others (Emery, 2002).

Self-renewal of satellite cells is the primary mechanism responsible for maintaining a viable satellite cell pool (Collins et al., 2005; Montarras et al., 2005; Sacco et al., 2008). Pax7 is expressed by quiescent satellite cells and is implicated in the generation of committed myogenic progenitors (Lepper et al., 2009; Seale et al., 2000). Satellite cells are normally mitotically quiescent in adult and so must first be activated to proliferate, in order to generate myoblasts that eventually differentiate to provide myonuclei to repair myofibres. Following activation, satellite cells co-express Pax7 with MyoD and then undergo their first cell division. Later, satellite cell-derived myoblasts either down-regulate Pax7, maintain MyoD and induce myogenin as they differentiate, or down-regulate MyoD and maintain Pax7, returning to a quiescent-like state (Halevy et al., 2004; Zammit et al., 2004b). Such Pax7^+^/MyoD^−^ cells also exhibit an increase in sphingomyelin levels (Nagata et al., 2006a; Nagata et al., 2006b) and re-expression of a Nestin transgene (Day et al., 2007), both of which are hallmarks of quiescent satellite cells.

Here we first investigated the range of functional properties of satellite cells and then determined whether they were different in cells with separate embryonic origins in both young and old mice. We chose the MAS as an example of a branchiomeric muscle, and compared the properties of its satellite cells to those from the extensor digitorum longus (EDL) of the limb, derived from somites. Satellite cell-derived myoblasts from MAS and EDL muscles had distinct gene expression profiles, and satellite cells of limb muscles made a cell fate decision to either self-renew or differentiate earlier than those from the MAS, which were generally more proliferative. However, MAS-derived satellite cells were able to regenerate limb muscles with equal efficiency to limb-derived cells after grafting. Clonal analysis revealed that satellite cells from muscles of both embryonic origin contained cells with a continuum of properties, with respect to their proliferative capacity and differentiation efficiency. Some satellite cell clones proliferated extensively and gave rise to many self-renewed and differentiated progeny, while other clones differentiated completely. In general though, MAS-derived satellite cells were more proliferative and made a cell fate decision later. In old muscle, functional heterogeneity remained in satellite cells from both limb and head muscles, although there was a general loss of proliferative ability, especially in the MAS. Therefore, satellite cells exist as a functionally heterogeneous population in both somitic and branchiomeric muscles, with many occupants of the satellite cell niche exhibiting stem cell characteristics.

## Materials and methods

### Cell culture

Mice were bred, and experimental procedures were carried out, under the provisions of the Animals (Scientific Procedures) Act 1986. Adult male C57BL10 (8–12 weeks old) were used for all in vitro experiments on young mice, *Myf5*^*nlacZ*^^/*+*^mice (Beauchamp et al., 2000) were used for in vitro experiments on old mice (> 2 years old) and *3F-nlacZ-E* mice for transplantation studies. Mice were killed by cervical dislocation, and muscles dissected and digested in collagenase, as described in detail elsewhere (Collins and Zammit, 2009).

For non-adherent cultures, myofibres and associated satellite cells were cultured in plating medium (DMEM supplemented with 10% horse serum, 0.5% chicken embryonic extract, 4 mM l-glutamine and 1% penicillin/streptomycin) at 37 °C in 5% CO_2_ as previously described (Zammit et al., 2004b).

For studies of differentiation efficiency, freshly isolated myofibres were mildly digested with 0.125% trypsin–EDTA for 10 min at 37 °C, and the released satellite cells were plated on Matrigel in 75 cm^2^ flasks or 6-well plates and maintained at low density for several days in DMEM supplemented with 20% FBS, 1% chicken embryo extract, 4 mM l-glutamine and 1% penicillin–streptomycin. Cells were then trypsinised and counted using a haemocytometer and re-plated at the same density per well in Lab-Tek 8-well chamber slides for each muscle and further cultured before immunostaining.

For clonal studies, a clean isolated myofibre was incubated in 0.125% trypsin–EDTA (Sigma) for 10 min at 37 °C in a microtube, to mildly digest the myofibre and liberate the associated satellite cells. The entire contents of the tube were then mixed well and evenly plated into two Matrigel coated 8-well chamber slides (Lab-Tek) so that a total surface area of 12.8 cm^2^ was used per myofibre (0.8 cm^2^ per well × 8 wells × 2 slides). Cultures were maintained in DMEM supplemented with 20% FBS, 1% chicken embryo extract, 4 mM l-glutamine and 1% penicillin–streptomycin. Theoretically we should have plated a single cell per well, in addition to plating at clonal density. In the vast majority of cases a single colony was found per well as expected. Rarely, two well-separated colonies were found in a single well, in which case they were treated as having arisen from separate satellite cells. The number of cells per colony was counted directly under the microscope for small colonies. For big colonies, images were collected of the entire colony and cell number was determined by counting from a computer monitor or using “ImageJ.”

### Immunocytochemistry

The following antibodies were used: rat monoclonal anti-Ki67 (Dako Ltd., Kyoto, Japan), rabbit polyclonal anti-MyoD (Santa Cruz Biotechnology, Santa Cruz, CA), mouse monoclonal anti-MyHC (MF20), anti-Pax7 and anti-myogenin (F5D) (DSHB, Iowa City, IA).

Immunocytochemistry was performed as previously described (Ono et al., 2007). After fixation with 4% PFA, cells were incubated with primary antibodies at 4 °C overnight and the primary antibodies visualised using appropriate species-specific 488 and 594 fluorochrome-conjugated secondary antibodies (Invitrogen) and mounted in Vectashield fluorescent mounting medium containing 4,6-diamidino-2-phenylindole (DAPI) (Vector Laboratories, Burlingame, CA). A Zeiss Axiophot 200 M microscope using Plan-Neofluar lenses was used to acquire digital images using a Zeiss AxioCam HRm and AxioVision software version 4.4 (Zeiss). For statistical analysis, Student's T-test was used, where results were deemed significant if *p* < 0.05.

### Quantitative RT-PCR

Myofibres were isolated, then plated at ∼ 150/well on Matrigel coated 6-well plates and cultured in plating medium for 72 h. Myofibres were then removed and myoblasts cultured for a further 24 h in DMEM supplemented with 30% FBS, 0.5% chick embryo extract, 10 ng/ml bFGF, 4 mM l-glutamine and 1% penicillin/streptomycin. Total RNA was extracted using the RNeasy Kit (Qiagen) and cDNA prepared from 250 ng of RNA with the QuantiTect Reverse Transcription Kit with genomic DNA wipeout (Qiagen). QPCR was performed on an Mx3005P QPCR system (Stratagene) with Brilliant II SYBR green reagents and ROX reference dye (Stratagene) (Collins et al., 2009). Primers for *Gapdh* (normaliser), *Pax3*, *Pax7*, *Myf5*, *MyoD*, *Pitx1*, *Pitx2a*, *Pitx2b* and *Pitx2c* were as described previously (Boerboom et al., 2006; Collins et al., 2009; Martinez-Fernandez et al., 2006). Primers for *Lbx1* (F 5′-CCGCCCGCCAACTCCAACAA-3′ and R 5′-GCGTCTGCCTCTGCCCAAAGA-3′), *Tcf21* (F 5′-ACATTCACCCAGTCAACCTG-3′ and R 5′-CCACTTCCTTCAGGTCATTCTC-3′), *Mrf4* (F 5′-TAAGGAAGGAGGAGCAAACG-3′ and R 5′-CCTGCTGGGTGAAGAATGTT-3′) and *Pitx3* (F 5′-GCTACCCTGACATGAGCACC-3′ and R 5′-CCACCTTTGCACAGCTCC-3′) were designed using primer blast (NCBI). Relative expression between satellite cell-derived myoblasts from EDL and MAS was compared per mouse (*n* = 3 mice) and significance was determined using Student's T-test.

### Muscle transplantation

As a means to identify and locate donor-derived muscle, we used the *3F-nlacZ-E* transgene (Kelly et al., 1995), which has robust nuclear-localized β-galactosidase activity in myonuclei. We confirmed that MAS muscle, and myotubes produced from MAS-derived satellite cells *in vitro*, expressed this transgene (data not shown). The donor *3F-nlacZ-E* transgenic mice are on a wild-type background, and so express dystrophin at the periphery of skeletal myofibres and in the heart. In contrast, the host *mdx* mouse carries a point mutation in the *Dmd* gene that creates a premature stop codon (Bulfield et al., 1984), so its muscle only contains the occasional revertant myofibre with dystrophin expression. Therefore the contribution of donor satellite cells to muscle regeneration can be evaluated by counting the number of dystrophin-expressing myofibres in an area of engraftment as identified by the presence of X-gal^+^ myonuclei.

MAS and EDL myofibres of *3F-nlacZ-E* mice were isolated and the satellite cells were then physically dissociated from the myofibre by tituration using a needle as described elsewhere (Collins et al., 2005). After filtration through a 40 μm cell-sieve, cells were centrifuged and an aliquot was stained with trypan-blue. Small, round trypan-blue excluding cells were then directly counted on a haemocytometer. Approximately two hundred MAS-derived satellite cells were injected into the left, and ∼ 200 from the EDL into the right, tibialis anterior (TA) muscles of isofluorane anaesthetized 3-week-old *mdx*-nude mice (*n* = 7), whose hind limbs had been irradiated with 18 Gy 3 days previously. Engrafted muscles were removed 4 weeks later and multiple 7 μm transverse cryosections were collected at 100 μm intervals throughout the muscle. A cryosection from each level along the length of the muscle was first incubated in X-gal to reveal β-galactosidase activity in myonuclei derived from the donor *MLC3F* transgenic mouse, to identify and locate regions containing successful engraftment. Sections serial to those containing X-gal^+^ myonuclei were then immunostained for dystrophin (Collins et al., 2005). The contribution of donor satellite cells to muscle regeneration was evaluated by counting the number of dystrophin-expressing myofibres in sections containing the highest engraftment. One-way ANOVA test was performed to evaluate the significance of the data.

## Results

Cell behaviour was examined in satellite cells from three muscles with a somitic origin: the EDL and soleus (SOL) from the lower hindlimb, together with the extensor carpi radialis (ECR) of the forelimb. Satellite cell function was also examined in cells with a distinct embryonic origin: the jaw-closing MAS in the head, which is a branchiomeric muscle derived from cranial mesoderm.

### MAS contains significantly fewer satellite cells than limb muscles

**Fig. 1:**
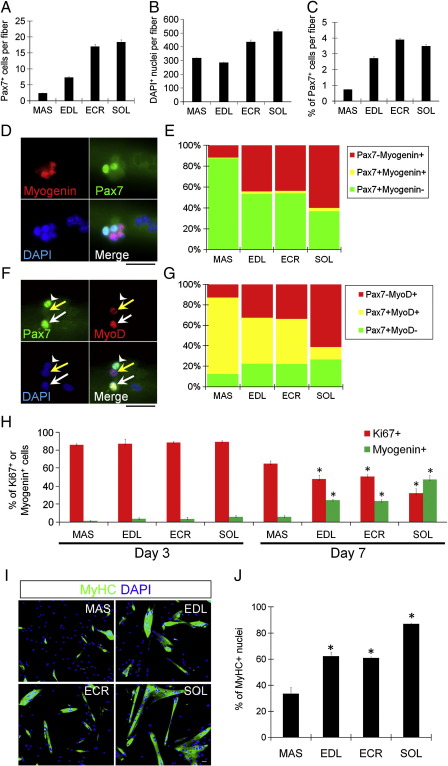
MAS-derived satellite cells proliferate more and differentiate later than those from limb muscles. Freshly isolated myofibres from the MAS, EDL, ECR and SOL were immunostained for Pax7, and all nuclei were visualised using a DAPI counter-stain. (A) The number of Pax7^+^ satellite cells, (B) number of DAPI^+^ nuclei and (C) percentage of total nuclei that are Pax7^+^ per individual MAS, EDL, ECR and SOL myofibre show that MAS has significantly fewer satellite cells than the other muscles—MAS (*n* = 63 from 3 mice), EDL (*n* = 91 from 4 mice), ECR (*n* = 75 from 3 mice) and SOL (*n* = 89 from 4 mice). (D and E) Satellite cells were cultured while in situ on their associated myofibre for 72 h and then co-immunostained for Pax7 and myogenin. There was a clear difference in commitment to myogenic differentiation, ranging from only ∼ 10% of MAS satellite cell progeny containing myogenin after 72 h in culture, compared to ∼ 60% for SOL. (F and G) Co-immunostaining parallel cultures for Pax7 and MyoD showed that there were also more cells with the self-renewing Pax7^+^/MyoD^−^ phenotype in the other muscles compared to MAS (Pax7^+^/MyoD^−^cells [arrowheads], Pax7^−^/MyoD^+^ cells [yellow arrows] and Pax7^+^/MyoD^+^cells [white arrows]. The low level of Pax7^+^/MyoD^+^ cells on SOL myofibres implies that the vast majority of SOL satellite cells have already made a fate decision by this time. (H) Myofibres were also digested to release the associated satellite cells, which were plated onto the tissue culture substrate and co-immunostained for Ki67 and myogenin after 3 or 7 days of culture. This showed that more MAS satellite cell progeny were still proliferating compared to the other muscles. (I and J) After 10 days, plated MAS-derived satellite cells exhibited less fusion into large multinucleated myotubes when immunostained for MyHC, compared to the other muscles even when initially plated at the same density. An asterisk denotes that data are significantly different compared to MAS (*p *< 0.05). Scale bars equal 30 μm.

Myofibres were isolated from the EDL, SOL, ECR and MAS, fixed and immunostained for Pax7 to identify satellite cells (Gnocchi et al., 2009). The mean number of satellite cells per myofibre was 2.4 ± 0.1 for MAS, 7.3 ± 0.4 for EDL, 17.0 ± 0.7 for ECR and 18.4 ± 0.7 for SOL (Fig. 1A). The total number of nuclei (satellite cells + myonuclei) per myofibre was 319.2 ± 3.5 for MAS, 285.8 ± 4.7 for EDL, 435.9 ± 14.3 for ECR and 512.4 ± 14.5 for SOL (Fig. 1B). Thus, even when the number of satellite cells per myofibre was expressed as a percentage of total nuclei, the MAS still had significantly fewer than the other muscles (0.74% ± 0.03 for MAS versus 2.72% ± 0.12 for EDL, 3.95% ± 0.09 for ECR and 3.62 ± 0.11 for SOL) (Fig. 1C).

### MAS-derived satellite cells differentiate later than those from limb muscles

To determine if satellite cells from different muscles proliferate, differentiate and self-renew in a similar way, we cultured satellite cells in their niche on isolated myofibres from the different muscles for 72 h, and then immunostained them for Pax7/MyoD or Pax7/myogenin (Zammit et al., 2004b). This protocol allows the fate of satellite cells to be examined since satellite cell progeny with a Pax7^−^/MyoD^+^ or Pax7^−^/myogenin^+^ phenotype are committed to myogenic differentiation, while satellite cells that adopt a Pax7^+^/MyoD^−^ phenotype are expected to self-renew (Zammit et al., 2004b). The percentage of satellite cell progeny entering the myogenic differentiation pathway was significantly lower in MAS (Pax7^−^/MyoD^+^ 12.9%, Pax7^−^/myogenin^+^ 11.7%), compared to EDL (Pax7^−^/MyoD^+^ 32.6%, Pax7^−^/myogenin^+^ 44.1%), and ECR (Pax7^−^/MyoD^+^ 33.2%, Pax7^−^/myogenin^+^ 43.7%). There were many more differentiating cells in SOL (Pax7^−^/MyoD^+^ 60.8%, Pax7^−^/myogenin^+^ 60.0%), compared with the other muscles (Figs. 1D–G). More cells with the Pax7^+^/MyoD^−^ self-renewal phenotype were also found in the other muscles compared to MAS (Fig. 1G). Indeed the Pax7^+^/MyoD^+^ population in MAS was 74.8%, compared to only 12.3% for SOL, showing that the vast majority of MAS cells have not made a fate decision to self-renew or differentiate by this time (72 h) (Fig. 1G).

While culturing satellite cells attached to a myofibre provides an effective model to study the initial events of muscle regeneration, it is not suited to examine the later stages such as fusion into large multinucleated myotubes. Therefore, to further investigate the differentiation capacity of satellite cells from separate muscles, cells were removed from isolated myofibres by mild trypsinisation, and plated onto Matrigel and allowed to proliferate. They were then passaged, counted using a haemocytometer and plated at the same density in chamber slides to assess proliferation, differentiation and fusion by immunostaining for Ki67, myogenin, or myosin heavy chain (MyHC). A similar percentage of satellite cell-derived myoblasts from all four muscles were proliferating (Ki67^+^) when examined after 3 days in culture (Fig. 1H). After 7 days of culture, however, there were significantly more proliferating MAS-derived myoblasts, and fewer differentiating (myogenin^+^) ones, than found in satellite cell progeny from the other three muscles, with SOL-derived myoblasts again having the most extensive differentiation (Fig. 1H). After 10 days of culture, the percentage of MyHC^+^ cells and myotubes was lower in the MAS-derived myoblast cultures than those initiated from the EDL and ECR, and highest in those from SOL (Fig. 1I and quantified in Fig. 1J). To distinguish between a delay, or an inability, to effectively differentiate, MAS cultures were examined after 20 days, when they contained many large myotubes, showing that even though many cells enter the differentiation program later, they are still able to efficiently differentiate (data not shown). Taken together, these results showed that satellite cells are intrinsically different between muscles: there are many more satellite cells from SOL muscle (∼ 9 fold more than MAS) which differentiate efficiently, while MAS myofibres not only have fewer satellite cells, but they are generally more proliferative and enter differentiation later than those from EDL, SOL and ECR.

### Gene expression profiles differ between satellite cell-derived myoblasts from EDL and MAS

**Fig. 2:**
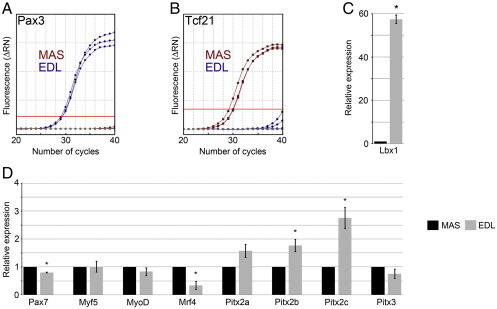
Comparison of gene expression profiles of EDL and MAS satellite cell-derived myoblasts. QPCR analysis was performed on proliferating satellite cell-derived myoblasts from the EDL and MAS. (A and B) Amplification plots of *Pax3* levels show that there was virtually no expression in MAS-derived myoblasts compared to those of the EDL, while conversely, *Tcf21* was robustly expressed in myoblasts of the MAS but not EDL. (C) Relative expression of *Lbx1* was approximately ∼ 57 fold higher in the EDL-derived myoblasts compared to those of the MAS. (D) Analysis of the relative expression levels showed that *Pax7* was lower in myoblasts of the EDL than MAS, while of the myogenic regulatory factors, only *Mrf4* was significantly different, being lower in EDL myoblasts. *Pitx2* isoforms *Pitx2b* and *Pitx2c* were significantly higher in EDL-derived myoblasts compared to MAS (for *Pitx2a p *= 0.06). Analysis performed on myoblasts from three mice, where an asterisk denotes that data are significantly different (*p *< 0.05).

For further experiments, we used just the EDL and MAS, since they have similar fibre type compositions: EDL contains ∼ 90% IIx and IIb (Rosenblatt and Parry, 1992), and MAS is composed of ∼ 95% fast type IIx and IIb fibre types (Muller et al., 2001). We first used QPCR to compare the gene expression profiles of proliferating satellite cell-derived myoblasts (after 4 days of culture) from the EDL and MAS (Fig. 2). Considering the central role that Pax3 and Pax7 play in myogenesis, we first examined their expression. Measurement of *Pax3* mRNA demonstrated that it was virtually undetectable in MAS-derived myoblasts, but robust expression was found in those from EDL (Fig. 2A). Conversely *Tcf21*, an essential transcriptional factor for craniofacial muscle formation (Lu et al., 2002), was robustly expressed in myoblasts of the MAS but not EDL (Fig. 2B). As expected, relative expression of *Lbx1*, which is required for limb muscle formation (Schafer and Braun, 1999), was significantly higher (∼ 57 fold) in the EDL-derived myoblasts compared to those of the MAS (Fig. 2C). We then examined expression of the paired-like homeodomain transcription factor (*Pitx*) genes as they play a role in initiating the myogenic regulatory cascade during head muscle development (Dong et al., 2006; Shih et al., 2007) and also investigated the myogenic regulatory factor family. While *Pax7*, *Myf5*, *MyoD*, *Pitx2a*, *Pitx2b*, *Pitx2c* and *Pitx3* were all robustly expressed in satellite cell-derived myoblasts from both the EDL and MAS, *Pax7* and *Mrf4* levels were lower in myoblasts from the EDL (Fig. 2D). *Pitx2* isoforms *Pitx2b* and *Pitx2c* were significantly higher in EDL-derived myoblasts than MAS (*Pitx2a* only just failed to be significantly increased *p* = 0.06; Fig. 2D) and *Pitx1* levels were negligible in cells from both muscles (data not shown).

### Branchiomeric satellite cells regenerate somite-derived muscle

When limb-derived satellite cells are transplanted into the irradiated TA muscle of a *mdx*-nude mouse, they can generate hundreds of new myofibres (Collins et al., 2005). Since satellite cells from the MAS have a different embryonic origin, and are generally more proliferative and differentiate later than somite-derived cells, we sought to establish whether they too could effectively regenerate limb muscle.

**Fig. 3:**
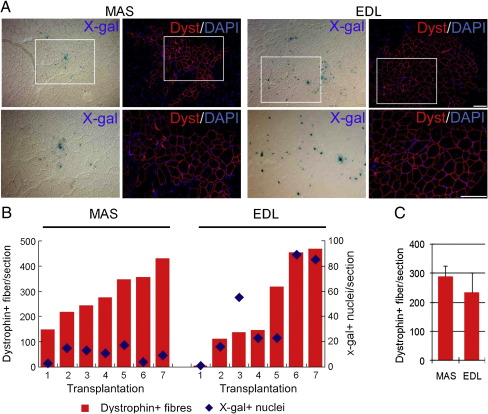
MAS-derived satellite cells efficiently regenerate hind limb muscle. Satellite cells were dissociated from isolated MAS and EDL myofibres of *3F-nlacZ-E* mice and ~  200 MAS satellite cells were injected into the left, and 200 EDL into the right, pre-irradiated TA muscle of recipient *mdx*-nude mice. (A) The contribution of these satellite cells to regeneration in the host muscle was revealed by using X-gal to localise the β-galactosidase activity of donor-derived myonuclei on cryosections, and then immunostaining serial sections for dystrophin to evaluate the extent of the donor cell contribution to regenerated myofibres. All 14 TA muscles grafted with either ∼ 200 MAS- or EDL-derived satellite cells had zones of donor-derived muscle. A region of TA muscle engrafted with either MAS-derived or EDL-derived satellite cells is illustrated in (A) with the area delimited by a white box shown in higher power. (B) The extent of donor-mediated muscle regeneration was quantified in each TA injected with MAS-derived (*n* = 7) or EDL-derived satellite cell (*n* = 7), and the highest number of dystrophin^+^ donor myofibres (red) shown, with the corresponding number of myonuclei with β-galactosidase activity (blue) also recorded. (C) The mean ± SEM number of dystrophin^+^ muscle fibres/section for MAS and EDL grafts were not significantly different (as analysed using one-way ANOVA). Scale bars equal 50 μm.

Satellite cells were isolated from the MAS and EDL of *3F-nlacZ-E* mice (*n* = 8) and 200 cells were grafted into the left and right irradiated TA muscles respectively of *mdx*-nude mice (*n* = 7). Grafted muscles were removed 4 weeks later and multiple transverse sections were taken at regular intervals along the length of the muscle. To identify and locate regions of the muscle with successful engraftment, cryosections were incubated in X-gal to reveal β-galactosidase activity from the donor-derived *MLC3F* transgene. Cryosections adjacent to those demonstrating engraftment by the presence of X-gal^+^ myonuclei were then immunostained for dystrophin (Fig. 3A). The contribution of donor satellite cells to muscle regeneration was evaluated by counting the number of dystrophin-expressing myofibres in these areas. All grafts contributed to muscle regeneration, with MAS-derived satellite cells making a mean ± SEM of 288.7 ± 36.2 (range 150–430) dystrophin^+^ myofibres, compared to a mean ± SEM of 234.6 ± 68.1 (range 5–470) for control EDL-derived satellite cells (Figs. 3B and C). Therefore both the proportion of grafts that generated new muscle and the amount of donor-derived dystrophin^+^ myofibres formed were not significantly different between muscles transplanted with either EDL-derived or MAS-derived satellite cells.

### Clonal studies of satellite cells

**Fig. 4:**
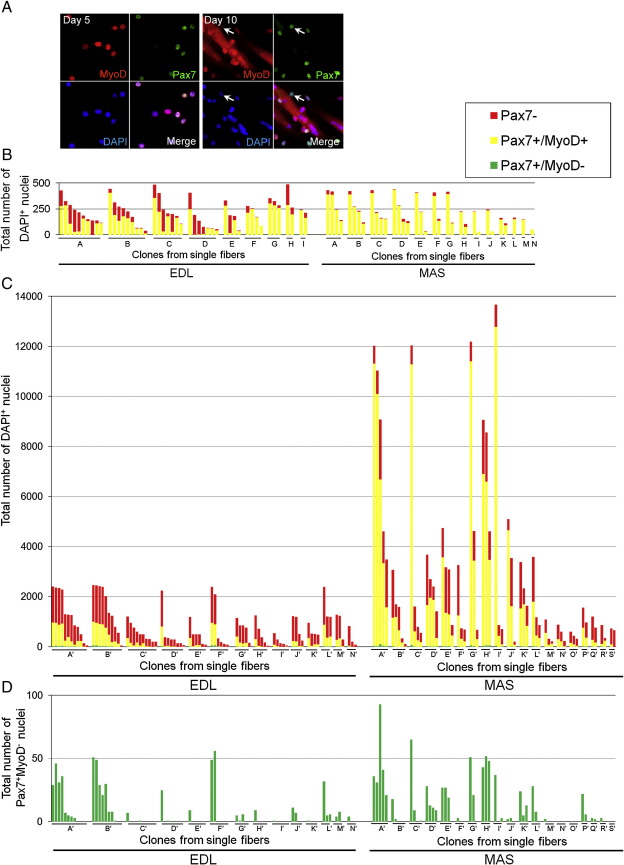
Functional heterogeneity between satellite cells associated with a single myofibre. Clonal analysis was performed from all satellite cells associated with an isolated myofibre from either EDL or MAS muscles. (A) Satellite cells isolated from individual myofibres were cultured and clones were analysed after 5 or 10 days by co-immunostaining for Pax7 and MyoD, with typical cultures illustrated (arrow indicates a Pax7^+^MyoD^−^ cell). (B and C) Expressing the individual clone data on a per myofibre basis (each letter on the X axis is an individual myofibre) shows the range of proliferative and myogenic abilities of distinct clones from the same myofibre after both (B) 5 days (EDL: 48 colonies from 9 myofibres A–I, MAS: 34 colonies from 14 myofibres A–N) and (D) 10 days (EDL: 84 colonies from 14 myofibres A'–N', MAS: 60 colonies from 19 myofibres A'–S'). Satellite cell clones from both EDL and MAS exhibited a large range in proliferative ability (smallest was 40, largest was 2468 cells/colony in EDL and 117–13,675 for MAS). (D) Due to the scale needed to include all cells in each clone in (C), it is difficult to see the number of Pax7^+^/MyoD^−^ cells, so these are also presented separately for each clone in (D).

To further examine the properties of satellite cells within a muscle, we next performed clonal analysis of each satellite cell associated with a particular myofibre. To be able to sample the entire satellite cell population, we isolated myofibres by our standard protocol using collagenase digestion (Collins and Zammit, 2009), but then separately protease-digested individual myofibres to release all the satellite cells associated with that myofibre, which were then cultured in 8-well chamber slides at clonal density. By keeping the culture conditions constant, we aimed to examine intrinsic differences between individual satellite cells. Clonal cultures were then co-immunostained for Pax7 and MyoD after 5 or 10 days (immunostaining of a typical clone illustrated in Fig. 4A).

### Satellite cells from the EDL are heterogeneous

**Fig. 5:**
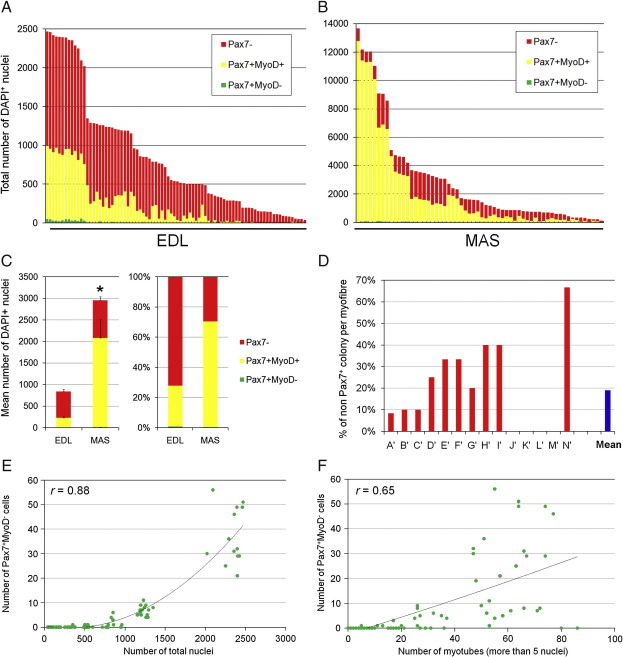
Self-renewal ability correlates with increasing clone size and number of myotubes. Re-ordering the data of Fig. 4C by clone size clearly illustrates the range in proliferative capacity in both (A) EDL-derived and (B) MAS-derived satellite cell cultures. (C) Combining data from all clones from the EDL and MAS to produce a population mean ± SEM highlights the significantly smaller mean clone size of EDL-derived satellite cells (842.5 ± 82.0) compared to those of the MAS (2960.3 ± 451.9) and the higher proportion of EDL myoblasts committing to differentiation than in MAS cultures. (D) Percentage of satellite cell clones per EDL myofibre (A'–N') that differentiated completely (non-Pax7^+^ colony) after 10 days from Fig. 4C, with a population mean of 19% clones per myofibre. (E and F) Relationship between the number of Pax7^+^/MyoD^−^ cells with a self-renewal phenotype in individual colonies and (E) the total clone size and (F) the number of myotubes (more than five myonuclei) shows that there is a positive correlation between the presence of self-renewing cells and both increasing clone size and myotube number. An asterisk denotes that data are significantly different (*p *< 0.01).

To demonstrate the range of satellite cell behaviour in individual clones, the results are presented on a “per myofibre” basis: all satellite cells from an individual myofibre were directly compared for phenotype and proliferative ability (Figs. 4B–D). There was a large range in proliferative potential between satellite cell clones from the same myofibre. For example, EDL-derived myofibre B' was associated with 10 satellite cells, the most proliferative of which gave rise to a colony of 2468 cells (∼ 11–12 doublings) after 10 days in culture, while the least proliferative clone from the same myofibre produced only 51 cells (∼ 5–6 doublings) (Fig. 4C). Expressing the data from the most to least proliferative clone, irrespective of their fibre of origin, the range for satellite cells clones from EDL muscle was 2468 to 40, a ∼ 62-fold difference (Fig. 5A). The mean ± SEM clone size for the EDL was 842.5 ± 82.0 (Fig. 5C).

After 10 days in culture it was also clear that the number of cells undergoing myogenic differentiation varied between satellite cell clones derived from the EDL. The majority of EDL-derived satellite cell clones contained Pax7^+^/MyoD^+^ cells, with some also having the self-renewing Pax7^+^/MyoD^−^ phenotype (Figs. 4D and 5A). However, ∼ 19% were solely composed of Pax7^−^/MyoD^+^ cells, presumably all committed to myogenic differentiation (Fig. 5D), suggesting that not all satellite cells can self-renew.

### Self-renewal potential correlates with total clone size

Stem cells are characterised by their clonal capacity both to generate differentiated progeny and to undergo self-renewal, and the potential to self-renew can be associated with proliferative ability (Ema et al., 2005). Individual EDL-derived clones after 10 days of culture showed a positive correlation between the number of cells with the Pax7^+^/MyoD^−^ self-renewal phenotype and the total clone size (*r* = 0.88, Fig. 5E). There was also a positive correlation between the number of Pax7^+^/MyoD^−^ cells and the number of myotubes (≥ 5 nuclei, *r* = 0.65, Fig. 5F, and ≥ 10 nuclei, *r* = 0.70, data not shown). Thus, satellite cells within a muscle exist as a functionally heterogeneous population, with some having a high proliferative ability that is associated with high self-renewal capacity, indicating that the satellite cell pool contains cells with more stem cell properties.

### MAS-derived satellite cell clones show a greater range of proliferative ability

The clonal study of satellite cells from the MAS also showed a clear heterogeneity between individual cells (Figs. 4B–D). There was an even larger range in proliferative potential between satellite cell clones from MAS than EDL: after 10 days the largest clone was 13,675 cells (∼ 13–14 doublings) from myofibre I', while the other two satellite cell clones from the same myofibre contained only 869 and 705 cells (Fig. 4C). Expressing the data from most to least proliferative clone from all the myofibres the range from the MAS-derived clones was ∼ 117 fold (13,675 to 117) (Fig. 5B) with a mean ± SEM clone size of 2960.3 ± 451.9 (Fig. 5C). MAS clones generally contained a high percentage of Pax7^+^/MyoD^+^ cells, with a low proportion either entering differentiation or self-renewing (Figs. 4C and 5B and C). There was again a positive correlation between the number of cells with the Pax7^+^/MyoD^−^ self-renewal phenotype and the total clone size (*r* = 0.82), but unlike the EDL, no MAS-derived clones were found that only contained the Pax7^−^/MyoD^+^ phenotype at this time (Figs. 4C and 5B). Therefore, satellite cells are also functionally heterogeneous within a branchiomeric muscle, yet cells from the MAS are generally more proliferative and differentiate later than those of the EDL.

### Functional heterogeneity of satellite cells is preserved in old muscle

**Fig. 6:**
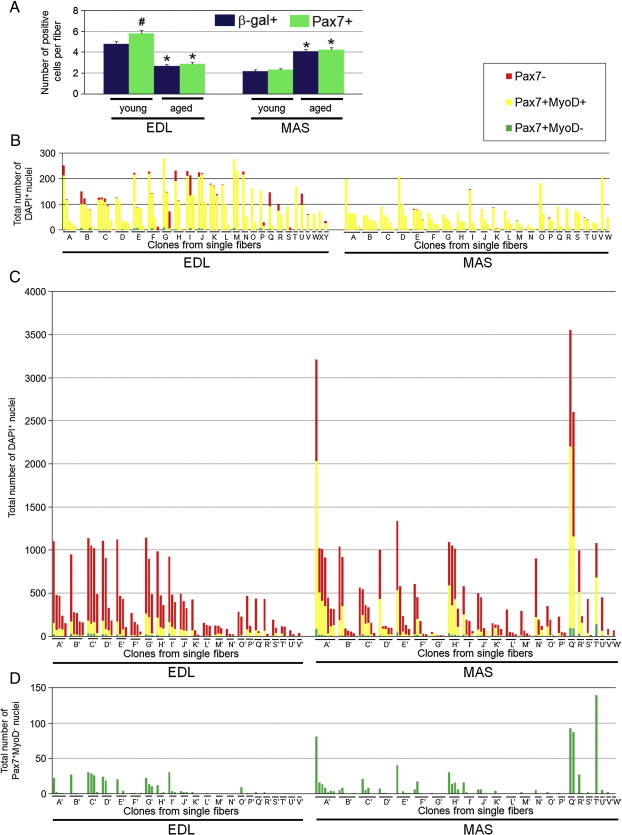
Satellite cell heterogeneity is preserved in both old EDL and MAS muscle. Satellite cell heterogeneity was also examined at the clonal level in aged (2 years+) mice. (A) Number of β-gal^+^ or Pax7^+^ satellite cells per myofibre from EDL (young *n* = 138, *n* = 117; old *n* = 91, *n* = 95, respectively) or MAS (young *n* = 110, *n* = 134, respectively; old *n* = 120 for both) fell with age in EDL muscle, but rose in MAS in *Myf5*^*nlacZ*^^/*+*^mice. (B) Co-immunostaining for Pax7 and MyoD of all satellite cell clones from an individual myofibre in EDL muscle (*n* = 66 colonies from 25 myofibres A–Y) or MAS muscle (*n* = 68 colonies from 23 myofibres A–W) in old *Myf5*^nlacZ/*+*^ mice was performed after 5 days of culture or (C) after 10 days culture (EDL muscle, *n* = 65 colonies from 22 myofibres A'–V' and MAS muscle *n* = 82 colonies from 23 myofibres A'–W'). (D) Due to the scale required to include all cells in each clone in (C), it is difficult to see the number of Pax7^+^/MyoD^−^ cells, so these are also presented separately for each clone in (D). An asterisk denotes that data are significantly different between young and old, while a sharp denotes a significant difference between β-gal^+^ and Pax7^+^ (*p *< 0.05).

Although the efficiency declines with age, the regenerative capacity of skeletal muscle remains throughout life, implying that satellite cells with stem cell characteristics must be retained (Collins et al., 2007; Conboy et al., 2005). We next asked how EDL and MAS satellite cells responded to aging, i.e., whether this distribution of satellite cell properties is also retained in old muscle, or if certain traits are preferentially lost. The *Myf5* locus is active in quiescent satellite cells (Beauchamp et al., 2000; Zammit et al., 2004a). The number of Pax7^+^ or β-gal^+^ cells per EDL myofibre was significantly lower in old *Myf5*^nlacZ/*+*^ mice (∼ 2 years old) than young (∼ 50% decline), but the number of satellite cells per MAS myofibre almost doubled in old mice (Fig. 6A), suggesting that a decrease in satellite cell numbers with age is a muscle-dependent process.

**Fig. 7:**
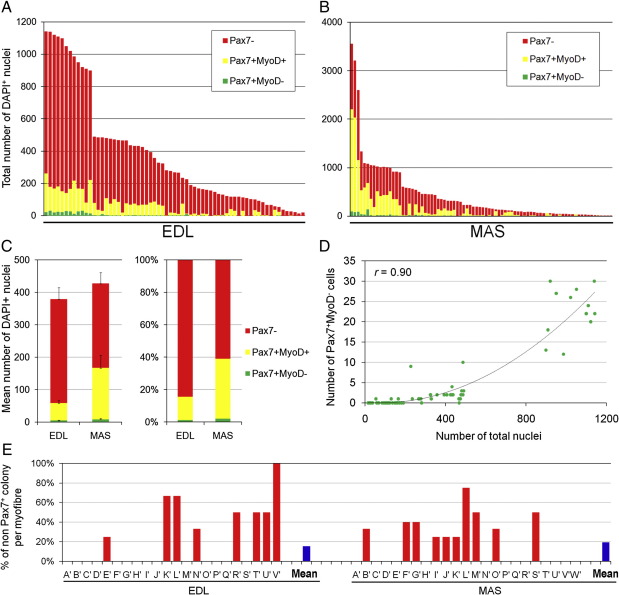
Satellite cell self-renewal correlates with size in clones from old mice. Re-ordering the data of Fig. 6C by clone size emphasizes the range of proliferative capacity in both (A) EDL-derived and (B) MAS-derived satellite cell cultures initiated from old mice. (C) Combining data from all clones from the EDL and MAS to produce a population mean ± SEM illustrates the similarity in clone size of EDL-derived satellite cells (379.1 ± 43.0) compared to those of the MAS (427.5 ± 70.4). A higher proportion of EDL-derived satellite cells from old mice differentiated earlier than cells from MAS. (D) As with young mice, there was again a strong positive correlation between the presence of the Pax7^+^/MyoD^−^ phenotype and increasing clone size in EDL. (E) In both EDL and MAS-derived satellite cell cultures there were a significant number of clones that did not contain any myoblasts expressing Pax7.

Unlike satellite cell clonal cultures initiated from young myofibres, a few colonies from old mice contained only Pax7^−^/MyoD^−^ fibroblast-like cells that did not make myotubes, so were excluded from the analysis. As with satellite cell clones from young mice (Figs. 4 and 5), satellite cell-derived clones from old muscle remained heterogeneous (Figs. 6B–D and 7A and B). The large range in satellite cell proliferative ability was retained in old muscle-derived clones examined after 5 and 10 days (compare Figs. 6B and C and 7A with Figs. 4 and 5). The distribution of satellite cell clones from the MAS was more markedly shifted towards smaller clones, when compared to cells from young muscles (compare Figs. 7B with 5B). After 10 days of culture, there was no significant difference between mean clone size of EDL and MAS cultures initiated from old mice (mean ± SEM was 379.1 ± 43.0 for EDL and 427.5 ± 70.4 for MAS; Fig. 7C). The proportion of satellite cell progeny with the Pax7^−^/MyoD^+^ phenotype of differentiating cells from both old EDL and MAS muscles was higher than in clones from young mice, which was particularly striking for the MAS (Figs. 7A–C). At this time, cells with the Pax7^+^/MyoD^−^ self-renewal phenotype were present in some colonies from both muscles (Figs. 7A–C). Again, there was a strong positive correlation between the number of Pax7^+^/MyoD^−^ cells and the total number of nuclei in clones from both old EDL (*r* = 0.90) and MAS (*r* = 0.78), indicating that bigger clones are more likely to contain cells with the self-renewal phenotype (Fig. 7D), as was observed with young mice (Fig. 5E). The proportion of clones that did not contain any myoblasts expressing Pax7 after 10 days in culture was 15.4% for EDL-derived clones and 19.5% for those initiated from MAS (Fig. 7E). Therefore, although functional heterogeneity within the satellite cell pool is retained throughout life, the proliferative potential is reduced, with more cells undergoing differentiation earlier.

## Discussion

In this study we set out to examine three aspects of satellite cell biology. Firstly, the range of functional properties exhibited by satellite cells resident within a particular muscle. Secondly, how satellite cell populations with distinct embryonic origins compare. And finally, how satellite cells with either a somitic or branchiomeric provenance respond to aging.

Satellite cell behaviour was examined in cells from the EDL, SOL and ECR muscles of somitic origin, and compared with those of the MAS in the head, derived from cranial mesoderm. Since it is now known that satellite cells share the ontogeny of the muscle that they reside in, then MAS-derived satellite cells have a different embryonic origin to those of the EDL, SOL and ECR (Armand et al., 1983; Harel et al., 2009; Schienda et al., 2006). There is evidence that satellite cells are heterogeneous between different somite-derived muscles, for example, with respect to their proliferation, differentiation dynamics and isoforms of MyHC expressed (Dusterhoft and Pette, 1993; Lagord et al., 1998; Rosenblatt et al., 1996; Rouger et al., 2004). We also found clear differences between the satellite cells derived from EDL, SOL and ECR; for example, SOL-derived cultures normally reached a fate decision to either self-renew or differentiate earlier than ECR or EDL. Compared to the three limb muscles, MAS had significantly fewer satellite cells, than even the EDL which has a similar fibre type composition (Muller et al., 2001; Rosenblatt and Parry, 1992). MAS-derived myoblasts proliferated more extensively than those of limb muscles and at 3 days of culture, ∼ 75% were still not expressing a phenotype consistent with either self-renewal or differentiation, compared to only ∼ 12% for SOL. Thus low numbers of satellite cells that proliferate longer before differentiation may contribute to the poor regenerative ability attributed to MAS in response to acute damage, compared to the efficient regeneration reported for limb muscle (Pavlath et al., 1998; Whalen et al., 1990).

Although MAS-derived satellite cells generally reached a cell fate decision later than those from the limb, they were able to differentiate efficiently and fuse into large myotubes when cultured for longer, showing that this extended proliferation did not compromise eventual differentiation. While transplantation assays have previously shown that jaw-closing muscle transplanted to limb can participate in muscle regeneration (Harel et al., 2009; Hoh and Hughes, 1991), there was little indication of how efficiently this occurred. Testing myogenic potential *in vivo* by grafting a small number (∼ 200) of EDL-derived satellite cells into one TA, and MAS-derived satellite cells into the TA of the other leg of the same host mouse, showed that MAS-derived satellite cells were able to generate dystrophin-expressing myofibres in host limb muscles as well as limb satellite cells themselves.

It has recently been shown that the molecular signature of quiescent satellite cells from the head and limb are different (Harel et al., 2009; Sambasivan et al., 2009). We found that proliferating satellite cell-derived myoblasts with separate embryonic origins also have different transcriptional profiles. For example, quiescent satellite cells from branchiomeric muscles express virtually no *Pax3* or *Lbx1*, but express *Tcf21* robustly (Harel et al., 2009; Sambasivan et al., 2009), differences that we found were retained in their myoblast progeny. We also examined *Pitx* genes, since these paired-like homeodomain transcription factors are expressed in developing muscle and are particularly important in head muscle formation (Grifone and Kelly, 2007; Shih et al., 2007). All *Pitx2* isoforms and *Pitx3* were highly expressed in proliferating satellite cell-derived myoblasts, although *Pitx2b* and *Pitx2c* levels were actually higher in cells from the EDL than MAS. This maintenance of distinct gene expression profiles in satellite cell-derived myoblasts suggests that even after activation and entry into the cell cycle, satellite cells from MAS and EDL retain an identity consistent with their ontogeny, which underlies their distinct properties.

In addition to examining the behaviour of populations of satellite cells from different muscles, we also performed clonal analysis to investigate the functional properties of individual satellite cells. To ensure that we only examined cells resident in the satellite cell niche *in vivo*, and also assayed representatives of the entire population (except those in muscle spindles), we first isolated myofibres and then clonally cultured all their associated satellite cells. Clonal cultures clearly demonstrated the functional heterogeneity of both EDL and MAS-derived satellite cell cultures. At one end of the spectrum were cells that underwent only a few rounds of cell division before all then differentiated, presumably corresponding to the cells that express myogenin mRNA within hours of muscle damage, and so commit to differentiation with little or no proliferation (Rantanen et al., 1995). At the other extreme are satellite cells that are capable of extensive proliferation, which also generated many cells with the Pax7^+^/MyoD^−^ self-renewal phenotype, as well as numerous differentiated progeny. Indeed, both clone size and the extent of differentiation were positively correlated to the presence of Pax7^+^/MyoD^−^ cells. A correlation between differentiation progeny and self-renewal has been noted for limb and diaphragm-derived satellite cells previously (Day et al., 2007), indicating that myotubes may well have a role in signaling to instruct other myoblasts to self-renew, rather than also differentiate. The satellite cell populations in both EDL and MAS therefore comprise both satellite cells with stem cell characteristics, together with others that may just produce limited numbers of differentiated progeny, consistent with previous observations in somite-derived muscles (Day et al., 2007; Schultz and Lipton, 1982; Shefer et al., 2006; Yablonka-Reuveni et al., 1987). The differences between satellite cell behaviour observed with bulk cultures from the EDL and MAS remained evident in the clonal studies, where MAS-derived clones generally proliferated more, and made a cell fate decision later, than those of the EDL.

Satellite cells can be potent myogenic progenitors: transplanting a single EDL myofibre (with a mean of ∼ 7 satellite cells) can not only generate substantial amounts of muscle, but also repopulate the host muscle with new satellite cells, indicating that there must have been extensive self-renewal of at least some of these few implanted cells (Collins et al., 2005; 2007). There is, however, great variation in the amounts of muscle and satellite cells produced by individual grafted myofibres, implying a difference in the properties of satellite cells associated with a particular myofibre (Collins et al., 2005; 2007). Recently, Sacco et al. (2008) have reported that grafting a single muscle stem cell can result in extensive proliferation (∼ 14–17 doublings), new muscle fibers and the re-isolation of donor-derived satellite cells. However, only 4% of single cells (3/72) successfully exhibited extensive expansion 4 weeks after transplantation, again implying that only a sub-population of satellite cells has stem cell properties.

Such functional heterogeneity in satellite cells begs the question of whether there are actually separate lineages. It has been known for some time that the *Myf5* locus is active in quiescent satellite cells (Beauchamp et al., 2000; Zammit et al., 2004a). Recently, Rudnicki and colleagues reported that there may be a satellite stem cell that can be identified by using recombination driven by *Cre* targeted to the *Myf5* locus (Kuang et al., 2007). These authors showed that approximately 90% of satellite cells had at one time expressed *Myf5*, since they expressed a recombination-activated reporter gene, while the remaining ∼ 10% did not. It was proposed that these *Myf5*-negative cells correspond to a dedicated subset of satellite cells that have more stem cell-like characteristics (satellite “stem” cells), with the remainder being their transit-amplifying progeny that undergo limited symmetric proliferation to generate myonuclei (Kuang et al., 2007). When *Cre* is targeted to the *MyoD* locus, however, essentially all quiescent satellite cells expressed a recombination-activated reporter gene, showing that they had expressed MyoD at some point (Kanisicak et al., 2009). It is perhaps more surprising that significant numbers of satellite cells with stem cell properties identified by never having activated the *Myf5* locus would have expressed *MyoD* at some point. It is also possible though that satellite cells start as a homogeneous population and over time, satellite cells might become a continuum of cells with more, or fewer, stem cell characteristics, perhaps because some cells have been activated fewer times, or have undergone less divisions [as has recently been proposed for skin stem cells (Clayton et al., 2007)], or are subject to different environmental signals (see Zammit, 2008 for a more detailed discussion).

We also explored how the aging process affects satellite cells. Old age is known to be associated with a significant decline in the mass, strength and regenerative capacity of skeletal muscles, suggesting that satellite cell function may be compromised (reviewed by Conboy and Rando, 2005). Several studies have shown that the number of satellite cells declines with age (e.g., Collins et al., 2007; Shefer et al., 2006). While EDL myofibres were indeed associated with ∼ 50% less satellite cells in our study though, the number of satellite cells in the MAS almost doubled in old mice, showing that a decline may not be a universal feature of muscle aging.

The large range in satellite cell proliferative ability between colonies, however, was retained in old muscles, although there was a significant decrease in the maximum and mean number of population doublings, consistent with earlier studies (Schultz and Lipton, 1982; Shefer et al., 2006). But this was more pronounced in the MAS-derived satellite cell clones though, with a loss of ∼ 3 mean population doublings, compared to ∼ 1 for EDL. This may be accounted for, in part, by the greater proportion of cells from both muscles that were entering myogenic differentiation earlier, and certainly in the case of the MAS, the significant number of colonies that did not contain any Pax7^+^ cells after 10 days in culture, a phenomenon not observed in clones from young mice. These observations show that the properties of the satellite cell population are directly affected by the aging process. Importantly, we again observed a strong positive correlation between the number of Pax7^+^/MyoD^−^ self-renewed cells and clone size from old donors, as with young. The maintenance of a population of satellite cells with stem cell characteristics in old muscle is consistent with our previous observations that old satellite cells grafted into young muscle remain capable of generating as much new muscle and satellite cells as grafting cells from young mice (Collins et al., 2007). Indeed a number of studies indicate that the old muscle environment is a major contributor to the inefficient regeneration observed (Carlson et al., 2001; Carlson and Faulkner, 1989; Collins et al., 2007; Conboy et al., 2005; Shefer et al., 2006). It has been reported that both structure and function in MAS muscles appear to be preserved with age though, compared to limb muscles (Norton et al., 2001). That the number of satellite cells increases in MAS muscles with age might be relevant to observations that satellite cell pool size could be an important factor for muscle maintenance (Shefer et al., 2006).

In conclusion, we have found that satellite cells from both somite-derived and branchiomeric muscles exist as a functionally heterogeneous population, and that many occupants of the satellite cell niche exhibit stem cell characteristics. Speculatively, satellite cells with different properties may be recruited for different tasks: for example, those that undergo limited proliferation may be geared towards performing homeostatic functions, requiring the occasional replacement of a few myonuclei. At the other end of the spectrum is muscle regeneration, requiring the synchronous activation and rapid expansion of the whole pool to generate many thousands of myonuclei, while also self-renewing to maintain a viable stem cell compartment with which to respond to future insult.
